# Multilevel regression modeling for aneuploidy classification and physical separation of maternal cell contamination facilitates the QF-PCR based analysis of common fetal aneuploidies

**DOI:** 10.1371/journal.pone.0221227

**Published:** 2019-08-20

**Authors:** Predrag Noveski, Marija Terzic, Marija Vujovic, Maja Kuzmanovska, Emilija Sukarova Stefanovska, Dijana Plaseska-Karanfilska

**Affiliations:** Research Center for Genetic Engineering and Biotechnology “Georgi D. Efremov”, Macedonian Academy of Science and Arts, Skopje, Republic of Macedonia; University of Helsinki, FINLAND

## Abstract

**Background:**

The quantitative fluorescent polymerase chain reaction (QF-PCR) has proven to be a reliable method for detection of common fetal chromosomal aneuploidies. However, there are some technical shortcomings, such as uncertainty of aneuploidy determination when the short tandem repeats (STR) height ratio is unusual due to a large size difference between alleles or failure due to the presence of maternal cell contamination (MCC). The aim of our study is to facilitate the implementation of the QF-PCR as a rapid diagnostic test for common fetal aneuploidies.

**Methods:**

Here, we describe an in-house one-tube multiplex QF-PCR method including 20 PCR markers (15 STR markers and 5 fixed size) for rapid prenatal diagnosis of chromosome 13, 18, 21, X and Y aneuploidies. In order to improve the aneuploidy classification of a given diallelic STR marker, we have employed a multilevel logistic regression analysis using "height-ratio" and "allele-size-difference" as fixed effects and "marker" as a random effect. We employed two regression models, one for the 2:1 height ratio (n = 48 genotypes) and another for the 1:2 height ratio (n = 41 genotypes) of the trisomic diallelic markers while using the same 9015 genotypes with normal 1:1 height ratio in both models. Furthermore, we have described a simple procedure for the treatment of the MCC, prior DNA isolation and QF-PCR analysis.

**Results:**

For both models, we have achieved 100% specificity for the marker aneuploidy classification as compared to 98.60% (2:1 ratio) and 98.04% (1:2 ratio) specificity when using only the height ratio for classification. Treatment of the MCC enables a successful diagnosis rate of 76% among truly contaminated amniotic fluids.

**Conclusions:**

Adjustment for the allele size difference and marker type improves the STR aneuploidy classification, which, complemented with appropriate treatment of contaminated amniotic fluids, eliminates sample re-testing and reinforces the robustness of the QF-PCR method for prenatal testing.

## Introduction

Prenatal testing for common fetal aneuploidies represents a genetic diagnostic procedure for the detection of abnormal number of chromosomes in fetal samples, obtained from amniocentesis, chorionic villus sampling (CVS) or percutaneous umbilical cord blood sampling (PUBS). C*ommon fetal aneuploidies* are usually referred to those resulting in live birth and include aneuploidies of chromosomes 13, 18, 21, X and Y, with trisomy 21 (Down syndrome) being the most common chromosomal disorder (1:800 live births) [1]. Beside the conventional karyotyping which is considered as the gold standard, there are several other genetic diagnostic methods for invasive aneuploidy analysis: fluorescence in situ hybridization (FISH), quantitative fluorescence PCR (QF-PCR), real-time quantitative PCR, digital PCR, mass spectrometry, multiplex ligation-dependent probe amplification (MLPA) and microarray-based comparative genomic hybridization (array CGH) [2,3]. Recently, with the emergence of the next-generation sequencing technologies, a noninvasive screening test for fetal aneuploidies has been introduced into clinical practice (non invasive prenatal testing, NIPT). This method is based on a quantitative analysis of the circulatingcell-free fetal DNA (cffDNA) fraction obtained with direct sequencing of the maternal plasmic DNA [4].

The QF-PCR for the testing of chromosomal aneuploidies was introduced in the early nineties with the emergence of automated capillary electrophoresis of fluorescently labeled PCR fragments [5,6]. This methodology enables the measurement of the fluorescence intensity of the amplified fluorescently labeled DNA fragments with variable lengths (short tandem repeats, STRs), in order to detect deviations from the normal 1:1 intensity ratio. The presence of three alleles with 1:1:1 ratio or two alleles with 2:1 or 1:2 ratio is indicative of trisomy/triploidy, while consistent monoallelic pattern of all tested markers for a given chromosome is indicative of monosomy in the analyzed samples. The clinical validity of QF-PCR for prenatal testing of common fetal aneuploidies has already been shown on large cohorts [7,8], with a clearly defined limitations for the detection of balanced and unbalanced chromosomal structural rearrangements, supernumerary markers and aneuploidies for other chromosomes [9,10,11].

The use of QF-PCR is proving to be a reliable and efficient method for the rapid prenatal diagnosis of fetal aneuploidy, and at the present time this method, together with the relatively new aCGH method (that can provide a much greater resolution than the conventional karyotyping), are perhaps the most used methods for invasive prenatal diagnosis. Furthermore, due to the presence of a certain percentage of false positive results in the NIPT analyses, the current recommendations include confirmation of all positive results with an invasive test [12,13], and QF-PCR is one of the most appropriate diagnostic tests for that purpose. Additionally, QF-PCR has the capacity to detect unusual genotypes, such as uniparental disomy [14].

Apart from the prenatal diagnostics, the QF-PCR analysis is applicable in many other fields. One of them is the detection of the most common genetic causes for male infertility associated with the existence of the microdeletions in the AZFc region of Y chromosome and the aneuploidies of the sex chromosomes; analysis, which has been introduced and conducted in our laboratory for a long period of time[15,16]. Additionally, we have used the QF-PCR method, more specifically the height ratio of the fixed-size marker amelogenin to analyze the association of the loss of Y chromosome (LOY) with the emergence of cancer [17], which, as a method for the detection of LOY, has already been applied by others[18]. This method of determining LOY in the blood, at least according to the obtained results for the existence of an association of LOY with different disease states is comparable to the SNP-Array method used for that purpose [19,20,21].

Despite the fact that QF-PCR has the advantage over other methodologies for being a rapid, robust and cost-effective diagnostic test which requires only a small amount of material and with high sensitivity and specificity [10], there are still some technical difficulties, mainly related to samples with large fragment size difference between alleles of a particular STR marker.

QF-PCR holds the advantage over the fluorescence in situ hybridization technique (FISH), regarding its capacity to directly detect maternal cell contamination (MCC) [22,23,24,25]. Nevertheless, the inability to resolve the inconclusive allele ratios due to the presence of the maternal genotype is the cause for the failure of the prenatal diagnosis.

The aim of our study was to address some technical difficulties in order to facilitate the implementation of the in-house QF-PCR as a prenatal diagnostic test for common fetal aneuploidies. We propose improvement of the aneuploidy classification of diallelic STR markers with the use of multilevel regression modeling. Furthermore, we present our experience with simple physical separation of the maternal blood cells from the fetal material cells in order to reduce the failure rate due to MCC. Here, we present an in-house, one-tube multiplex QF-PCR and diagnostic data from 4800 prenatal tests of common fetal aneuploidies.

## Materials and methods

### Subjects and sample testing

We have analyzed 4800 prenatal samples, 4235 of which were from the amniocentesis and 565 were from the CVS. Samples were collected and tested in a period of over sixteen years, during which the analysis was performed on three different genetic sequencers: ABI 310 (currently discontinued), ABI 3130 and ABI 3500 (Thermo Fisher Scientific, Waltham, Massachusetts, U.S, Foster City, CA, USA). Initially, we commenced the analysis with three multiplex QF-PCR reactions per sample, largely based on the work of Pertl et al. [26], followed by a combination of the markers into two reactions and ultimately we have created one single tube reaction. Of the 4800 samples, the first 1628 samples were analyzed with three multiplex QF-PCR reactions per sample, the next 698 samples were analyzed with two multiplex QF-PCR reactions per sample and the last 2474 samples were analyzed with one one-tube multiplex QF-PCR. The markers presented in this work, previously were used in different combinations and sometimes with a different dye labeling in the three and two multiplex PCR reactions. The one tube multiplex QF-PCR reaction was also changed over time, and therefore only the last 1355 fetal samples were analyzed with the one-tube PCR multiplex reaction presented in this work. All samples initially used for regression modeling (n = 871) and subsequently for testing of the prediction accuracy (n = 670) were obtained only from the cohort of subjects analyzed with the described one-tube PCR multiplex reaction. The percentages of detected aneuploidies and age distribution were calculated on a total number of pregnant women studied.

For the analysis of CVS and amniotic fluids with MCC, obtaining the maternal sample (blood or buccal swab) was obligatory, while for samples with clean uncontaminated amniotic fluid, the intention was to obtain a maternal sample in order to eliminate sample mix up. For samples positive for aneuploidy, we attempted to obtain a samples from the fathers, as well. In both cases we were able to obtain the majority of the intended samples (in more than 95% of the cases).

### Ethics statement

Oral informed consent was obtained from the participants in the study. From each participant personally we have recorded: age, gestational age and number of previous pregnancies. For the purpose of this research, all personal data were anonymized and the study was approved by the Ethics Committee of the Macedonian Academy of Sciences and Arts (09-1221/1).

### DNA extraction

DNA extractions of 0.5 to 5 ml amniotic fluids were performed, depending on the gestational week, cells present in the amniotic fluid and the total volume of amniotic fluid available and at least 2 chorionic villi. If the QF-PCR analysis had shown a contaminated chorionic villi with maternal DNA, subsequent multiple DNA extractions of single chorionic villi were performed.

Initially, the sample DNA was isolated with classical phenol/chloroform extraction and ethanol precipitation, but later (last 2344 samples) this method was substituted with the one-hour isolation protocol using the commercial High Pure Viral Nucleic Acid kit (Roche Applied Science, Mannheim, Germany). Parallel isolation of several consecutive batches of samples designated for prenatal diagnosis using phenol/chloroform extraction and High Pure Viral Nucleic Acid kit, showed that the later was comparable, and even better performing in cases with a low amount of amniocytes (grater total DNA yield and DNA concentration). DNA samples extracted using High Pure Viral Nucleic Acid kit showed better quality, i.e. more evenly distributed heights of the electrophoregram peaks. DNA concentration was measured using a Nanovue spectrophotometer (GE Healthcare, Little Chalfont, UK) and DNA quality was evaluated by measuring the ratios of absorbance at 260 nm/280 nm and 260 nm/230 nm.

### Quantitative fluorescence PCR reaction

**Fig 1:**
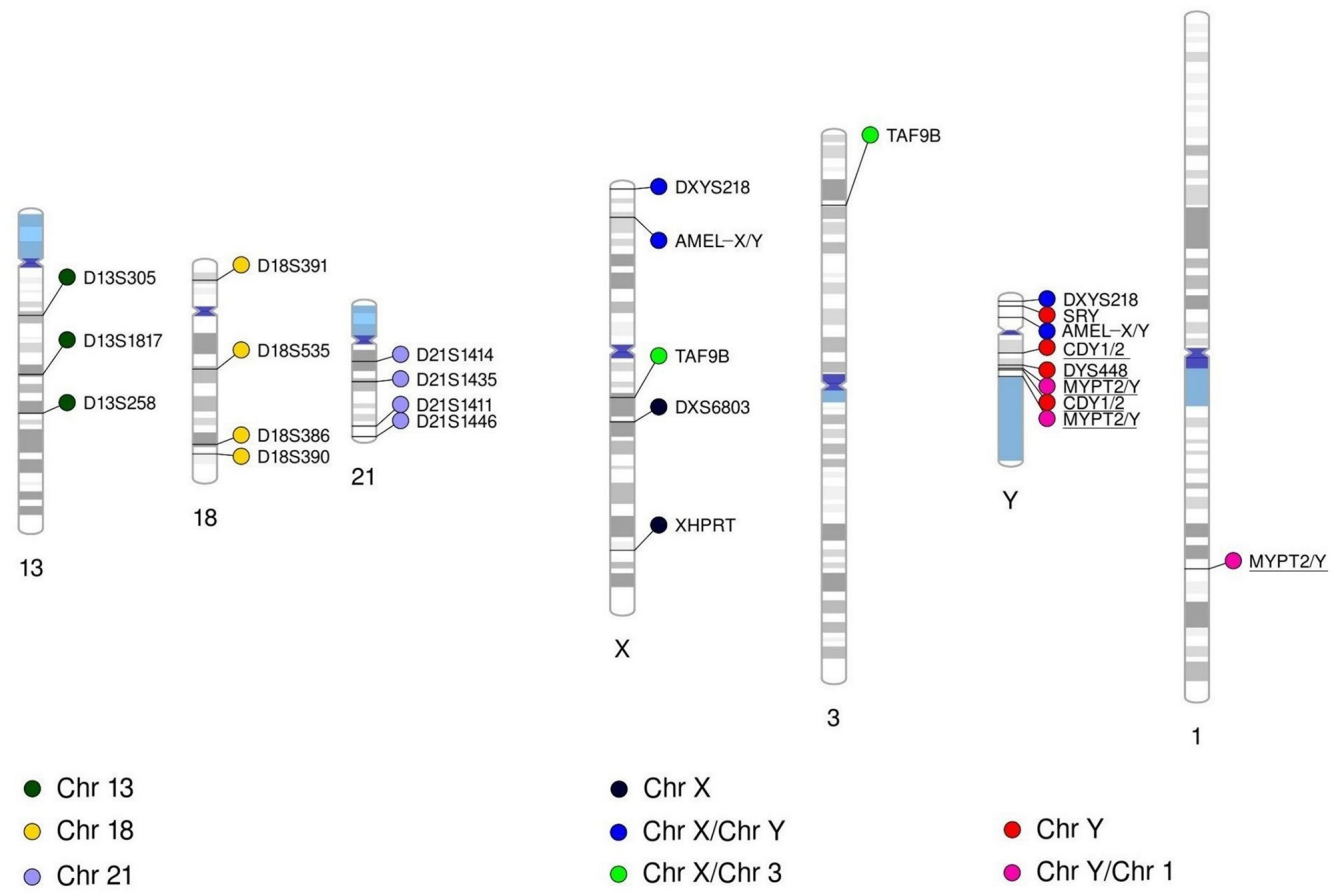
Chromosomal locations of the QF-PCR markers. The markers are colored by the chromosome or chromosomes they are designed to target. In order to distinguish between markers amplifying sequences on only one chromosome and markers amplifying sequences on different chromosomes simultaneously, we have organized a legend and coloring of the markers accordingly. Underlined markers are those used for detection of microaberrations in the AZFc region.

The one-tube QF-PCR multiplex consisted of 20 primer pairs (markers), of which the forward primer was fluorescently labeled, and they targeted in total 26 genomic positions (Fig 1). Of them, three markers were for analyzing the aneuploidies for chromosome 13, four for chromosome 18, four for chromosome 21 and six for the analysis of the sex chromosome aneuploidies. Two of the sex chromosome markers (DXS6803 and XHPRT) amplified sequences only on chromosome X (for counting chromosome X), two (AMELX/Y and DXYS218) co-amplified sequences of both X and Y chromosomes (for counting the X and Y chromosomes in male samples and the X chromosome in female samples), one (TAF9B) was for co-amplification of sequences on the chromosome X and 3 (for counting the X chromosome) and one (SRY) located on the Y chromosome was for male sex determination. Additionally, we included three markers (MYPT2/Y, DYS448 and CDY1/2) in the mix, in order to detect microaberrations in the azoospermia factor region “c” (AZFc), located on the Y chromosome. This region was previously related to male infertility, a complex phenotype being long-standing research interest of our group. Details about interpreting the results of the analysis with these three markers in the context of AZFc rearrangements are described elsewhere [15,16]. Marker MYPT2/Y co-amplifies sequences on chromosomes 1 and Y, therefore it is also used for confirmation of aneuploidy of the Y chromosome in male fetuses.

Details of the oligonucleotide sequences and other related data for the QF-PCR primers are given in S1 Table.

For cases with inconclusive results owing to the homozygosity of all markers for a given chromosome, we used additional QF-PCR reactions specific for the given chromosome. For chromosomes 13, 18 and 21, the primers were combined in one multiplex reaction each, while for the sex chromosomes we used additional, previously published multiplex reaction [16] for the detection of sex chromosome aneuploidies in infertile patients, and three markers as separate PCR reactions (for DXS6809, DXS996 and X22). The markers, oligonucleotide sequences and targeted genomic positions are shown in S2 Table and S1 Fig, respectively. As a second option for additional testing of cases with inconclusive results, especially when there was a larger number of monoallelic markers for a given chromosome, we used P-095 Aneuploidy MLPA kit (MRC-Holland, Amsterdam, Netherlands).

The multiplex reaction was performed in the volume of 20 μl consisting of 1X AmpliTaq Gold 360 Buffer (Thermo Fisher Scientific), 1.875 mM MgCl_2_, 187.5 μM each of the four dNTPs (dATP, dCTP, dGTP, and dTTP), 1.5 μl 360 GC Enhancer (Thermo Fisher Scientific), 50–667 nM primers, 0.75 U AmpliTaq Gold 360 DNA Polymerase (Thermo Fisher Scientific) and 1–50 ng DNA. The conditions for the PCR reaction were: 10 min at 95°C for the activation of the DNA polymerase, 29 cycles each consisted of 45 seconds at 95°C for DNA denaturation, 1 min. at 58°C for primer annealing and 1 min. 30 seconds at 72°C for elongation and finally one cycle of 30 min at 60°C for removing the stutter peaks. Although the guidelines of the European Cytogeneticists Association (ECA) recommend a number of PCR cycles between 24 and 26 for the reaction to remain in a semi-quantitative phase [27], 29 cycles were necessary for the successful amplification of all STR markers included in our assay, assessed by peak heights, at acceptable peak ratios. One μl of the finished reaction was combined with 12 μl of Hi-Di Formamide and 0.15 μl of GeneScan 500 LIZ Size Standard (Thermo Fisher Scientific) and run on ABI Genetic analyzer for automatic capillary electrophoresis. Data were analyzed using the GeneMapper analysis software version 4.0 and 4.1 (Thermo Fisher Scientific). Detailed protocol for performing DNA isolation from fetal material (amniotic fluid and chorionic villi), performing the main QF-PCR multiplex reaction and backup QF-PCR multiplex reaction for a given chromosome, together with electropherogram examples from normal and aneuploidic samples is presented at dx.doi.org/10.17504/protocols.io.2v9ge96 [28].

Although it may seem redundant, we would like to emphasize that two steps were critical for a successful multiplex PCR reaction: thorough dissolution of the DNA after its isolation and thorough homogenization of the DNA and PCR reaction mix prior the amplification on the thermal cycler.

### Multilevel regression modeling for aneuploidy classification of diallelic STRs

**Fig 2:**
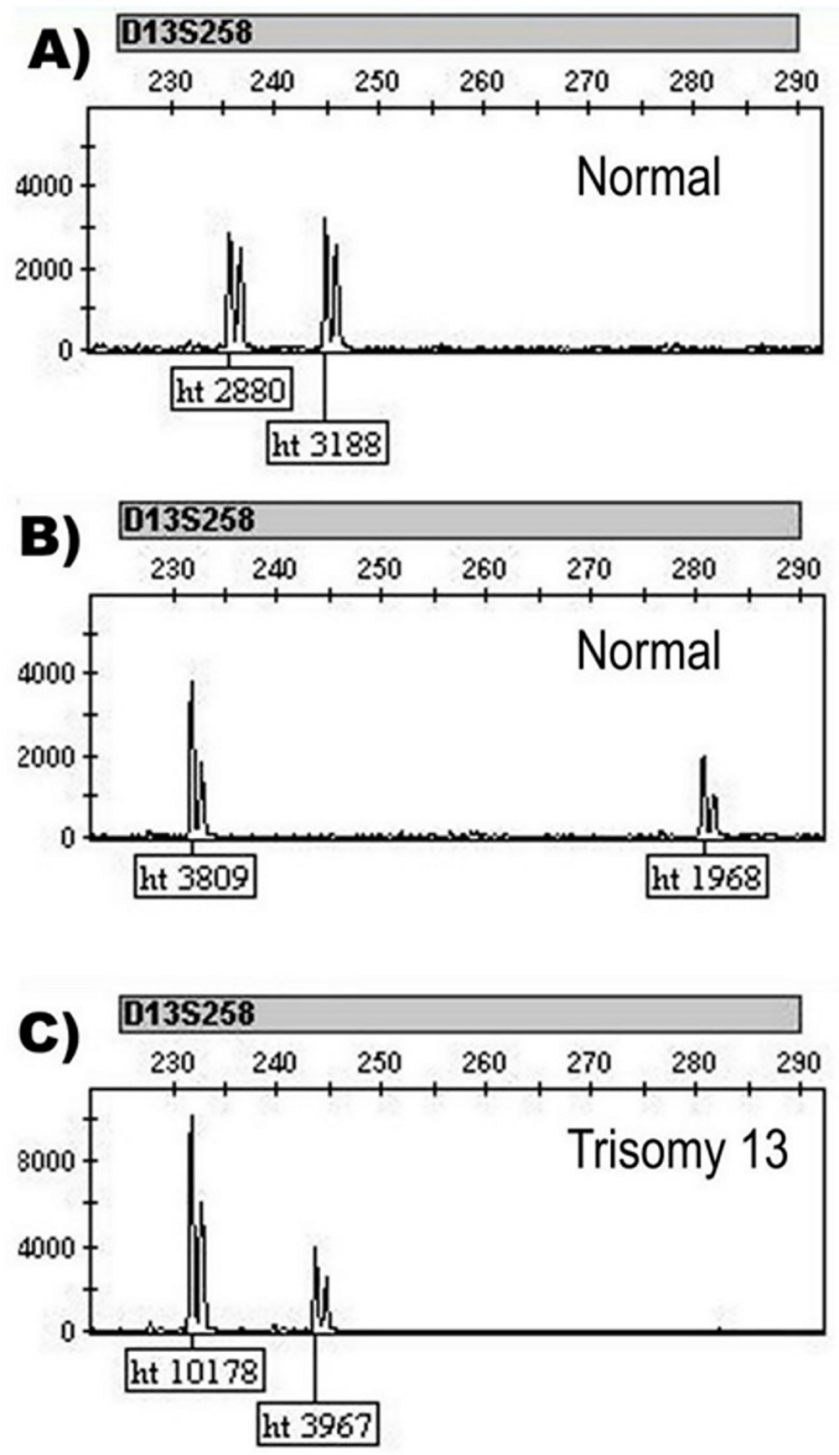
Electrophoregrams of three separate patients depicting variability of diallelic height ratios of the D13S258 STR marker. A) Height ratio of 0.90 from a sample without aneuploidy which deviates from the normal 1:1 ratio (usualy expected between 1–1.3 [29]); B) Height ratio of 1.94 from a sample without aneuploidy from which it is not possible to make a definite conclusion for the chromosome aneuploidy status (higher than 1.3 which is usually considered the upper limit of a normal finding [30]); C) As a comparison to a normal result, height ratio (2.57) for the same marker from a sample with trisomy 13 is given. Normality of the samples described under A and B and aneuploidy in the sample described under C was confirmed using MLPA analysis. The samples were run on ABI 3500 genetic analyzer and analyzed with GeneMapper 4.1.

When amplifying a large number of PCR fragments in a single reaction, there is variability of allele ratios which sometimes could substantially deviate from the normal 1:1 ratio (Fig 2A). Also, when using height ratio as an indicator of aneuploidy, sometimes there may be uncertainty regarding the result due to the large size difference between alleles, since the height ratio in patients without aneuploidy (Fig 2B) is comparable to that in patients with trisomy (Fig 2C). In order to improve the aneuploidy classification of a given diallelic STR marker we used multilevel regression modeling analysis using "height ratio" and "allele size difference" as fixed effects and "marker" as a random effect.

From a total of 871 samples (453 samples of fetal origin and 418 from parental origin) we have extracted 9104 diallelic peak height ratios for 14 STR markers excluding DYS448. Of them, 9015 were with normal 1:1 height ratio, 48 with trisomic 2:1 height ratio and 41 with trisomic 1:2 height ratio (S3 Table). The distribution of the numbers of the used ratios given by STR markers is presented in S2 Fig. Considering the fact that when evaluating the chromosome aneuploidy status with the use of diallelic peak height ratios, the positive result consists of two opposite values (theoretically 0.5 and 2) and the negative result is always around 1, we trained two regression models: one using diallelic 2:1 ratios, and a second one for 1:2 ratios. All 871 samples in this cohort used for the regression analysis were amplified under the same PCR conditions, run on ABI 3500 Genetic Analyzer and analyzed with GeneMapper 4.1 software. Height ratios were extracted using the GeneMapper 4.1 software. For some markers, for a given allele, two peaks were observable, caused by incomplete final extension with the extra "A" nucleotide, which is normally added by the Taq polymerase at the end of the PCR fragments. In those cases, the larger peaks were considered for computation of the height ratio.

In order to investigate the prediction accuracy of the regression model, prospectively, we used another set of 670 samples (327 of fetal origin and 343 of parental origin) of which we extracted 7002 diallelic peak height ratios for 14 STR markers excluding DYS448. Of them, 6967 were with a normal 1:1 height ratio, 19 with trisomic 2:1 height ratio and 16 with trisomic 1:2 height ratio (S4 Table). In this cohort we used samples which were run on two genetic analyzers: ABI3500 (5793 with normal 1:1 height ratio, 18 with trisomic 2:1 height ratio and 13 with trisomic 1:2 height ratio) and ABI3130 (1175 with normal 1:1 height ratio, 1 with trisomic 2:1 height ratio and 3 with trisomic 1:2 height ratio).

All samples with aneuploidy were confirmed with karyotyping or MLPA. Regarding euploidic samples, only a small cohort of 200 samples were confirmed with MLPA.

### Treatment of maternal cell contamination in amniotic fluids

In order to improve the outcome of the analysis of amniotic fluids defined as samples with MCC after visual inspection, we experimented and employed physical separation of the potentially contaminating material with the use of 6–12 hours of precipitation (S3 Fig). The precipitation was conducted only by storing the syringe with the amniotic fluid on +4°C. After the separation of the two clearly visible phases, first the upper clear phase was collected without dissolving the lower phase, after which the lower phase was collected separately. After centrifugation of the collected amniotic fluid, separate isolation and QF-PCR analysis were performed (S3 Fig). In order to accelerate the separation process, an alternative approach, with subsequent short time centrifugation of the contaminated amniotic fluid at low speed (500–1000 rpm) and followed by collection and separate analysis of the two segregated phases could give the similar effect.

### Statistical analysis

All statistical and data analyses were performed with the use of the R statistical software [31] if not otherwise stated. Data manipulation and creation of the graphics was performed using tydiverse v.1.2.1 library [32]. Multilevel regression modeling was performed with the lme4 v.1.1.15 library [33] using *glmer()* function with arguments family = *binomial* and link = *logit*. Calculation of the 95% confidence intervals for the estimated coefficients by regression analysis was performed with bootstrapping using *confint*.*merMod()* function from the lme4 library. Calculations and plotting of the results from the receiver operating characteristic (ROC) curve analysis was performed with pROC v.1.10.0 [34] and plotROC v.2.2.0 [35] libraries, respectively. Correlation between the two variables was analyzed using the Pearson correlation coefficient (r). Graphic illustrations of the chromosomal locations for the QF-PCR markers were created using PhenoGram [36].

## Results

### Success rates of one-tube multiplex QF-PCR for detecting trisomies 13, 18 or 21

From the last 1355 samples diagnosed with the presented one-tube multiplex PCR reaction, we have calculated the percentages of the samples homozygous for all tested markers of a given chromosome: 0.61% for the chromosome 13, 0.13% for the chromosome 18 and 0.13% for the chromosome 21. In the case of samples with the presence of only one heterozygous STR marker per chromosome (the other markers being homozygous), we observed one marker only heterozygosity in 8.49% of the samples for chromosome 13, 3.10% of the samples for chromosome 18 and 1.35% of the samples for chromosome 21. Using three (chromosome 13) instead of four (chromosomes 18 and 21) STR markers for establishing the diagnosis, increases the necessity for performing additional, chromosome specific QF-PCR reaction from 3 to 6 times. The difference(3.1% vs 1.35%) that is more than double between chromosomes 18 and 21 when it comes to the presence of only one heterozygous marker is due to the lower rate of heterozygosity of the markers D18S390 and D18S391 (0.681 and 0.685 respectively, S1 Table).

### Diagnostic data from aneuploidy testing

Of the 4800 analyzed samples, we could not issue a result for 122 samples due to maternal cell contamination (n = 114) and insufficient DNA material (n = 8). Of the remaining 4678 analyzed samples, 4495 had a normal result, while 182 samples showed a pattern consistent with aneuploidy: 97 with trisomy 21, 33 with trisomy 18, 15 with triploidy, 13 with trisomy 13, 10 with Klinefelter (XXY) syndrome, 9 with Turner (X0) syndrome, 3 with XXX syndrome, 1 with XYY syndrome and one double trisomic sample with trisomy 21 and Klinefelter syndrome.

**Fig 3:**
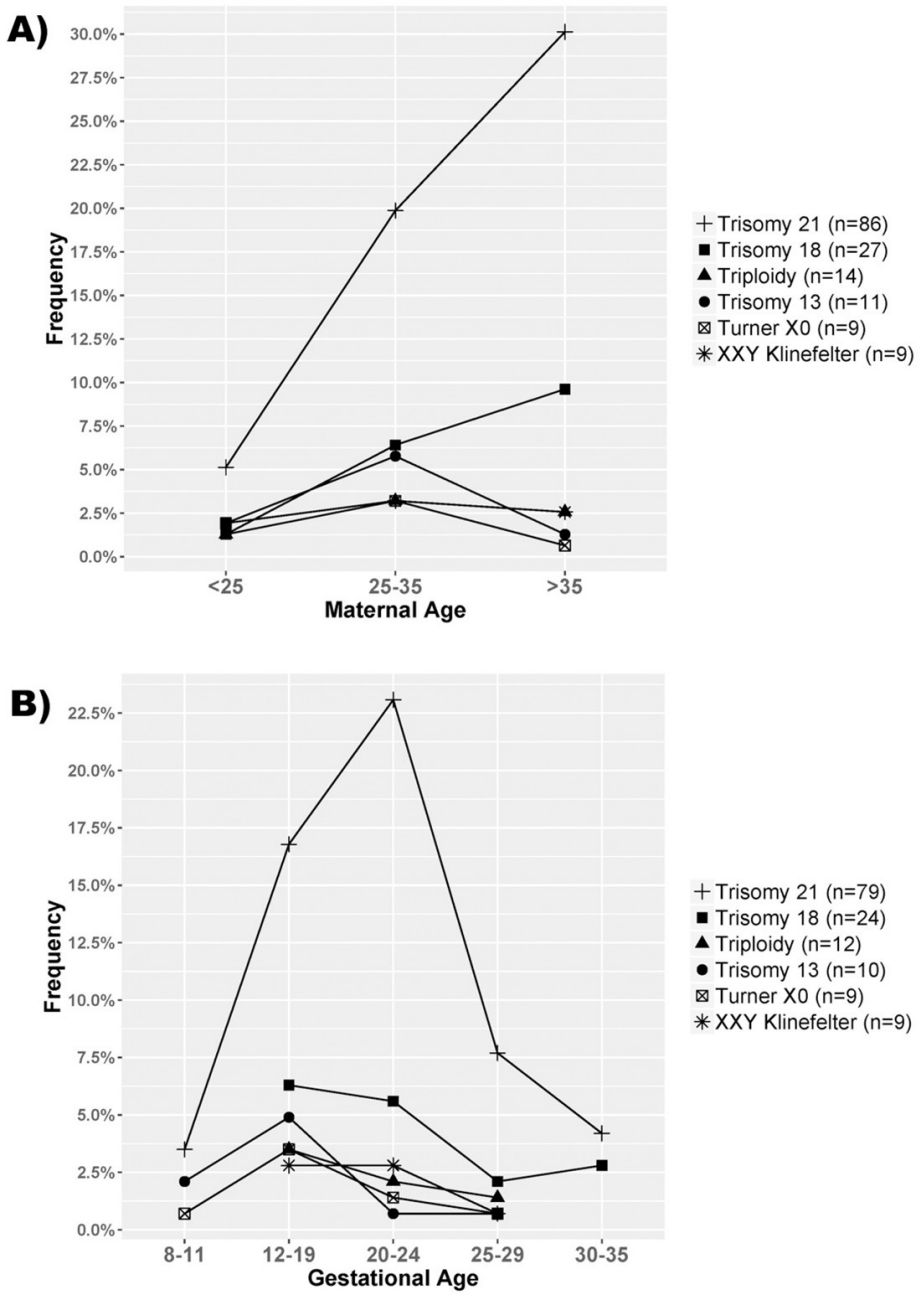
Graphical presentation of the proportions of maternal age and gestational age at diagnosis among the six most frequent types of aneuploidy. A) Distribution of the proportions of the maternal age aggregated in three different groups. The "y" axis shows the frequency of each group relative to the total number of aneuploidies (n = 156) with available data for MA. B) Distribution of the proportions of the gestational age aggregated in five different groups. The "y" axis shows the frequency of each group relative to the total number of aneuploidies (n = 143) with available data for GA.

We observed an increase in the frequencies of trisomy 21 and trisomy 18 with the increase of maternal age in contrast to other trisomies where there was a similar distribution of the frequencies between the three age groups (Fig 3A). Considering the gestational age at diagnosis, comparisons showed that trisomy 21 was detected with a slightly higher proportion towards the more advanced gestational period (later weeks of gestation, 20–24 weeks) as compared with other types of aneuploidies which were more frequently detected much earlier (20 weeks <) (Fig 3B). Detailed distribution of the counts of the maternal and gestational ages by different types of aneuploidies is given in S4 and S5 Figs respectively.

### Single markers with duplicated and de novo alleles

We detected duplicated alleles in nine samples for the DYXS218 STR marker, all of which were inherited from one of the parents and thus were considered as non pathogenic. Mutated alleles which differed in plus or minus one repeat from the mother’s alleles were observed for the D13S1817 (n = 2), D13S305 (n = 2), D13S258 (n = 1), D18S390 (n = 1), D18S386 (n = 1), DYXS218 (n = 1) and D21S1411 (n = 1) STR markers. The data for mutated alleles should be taken with caution and as incomplete because in most of the cases we have not tested the father of the referred samples.

### Multilevel regression modeling for aneuploidy classification of diallelic STRs

**Fig 4:**
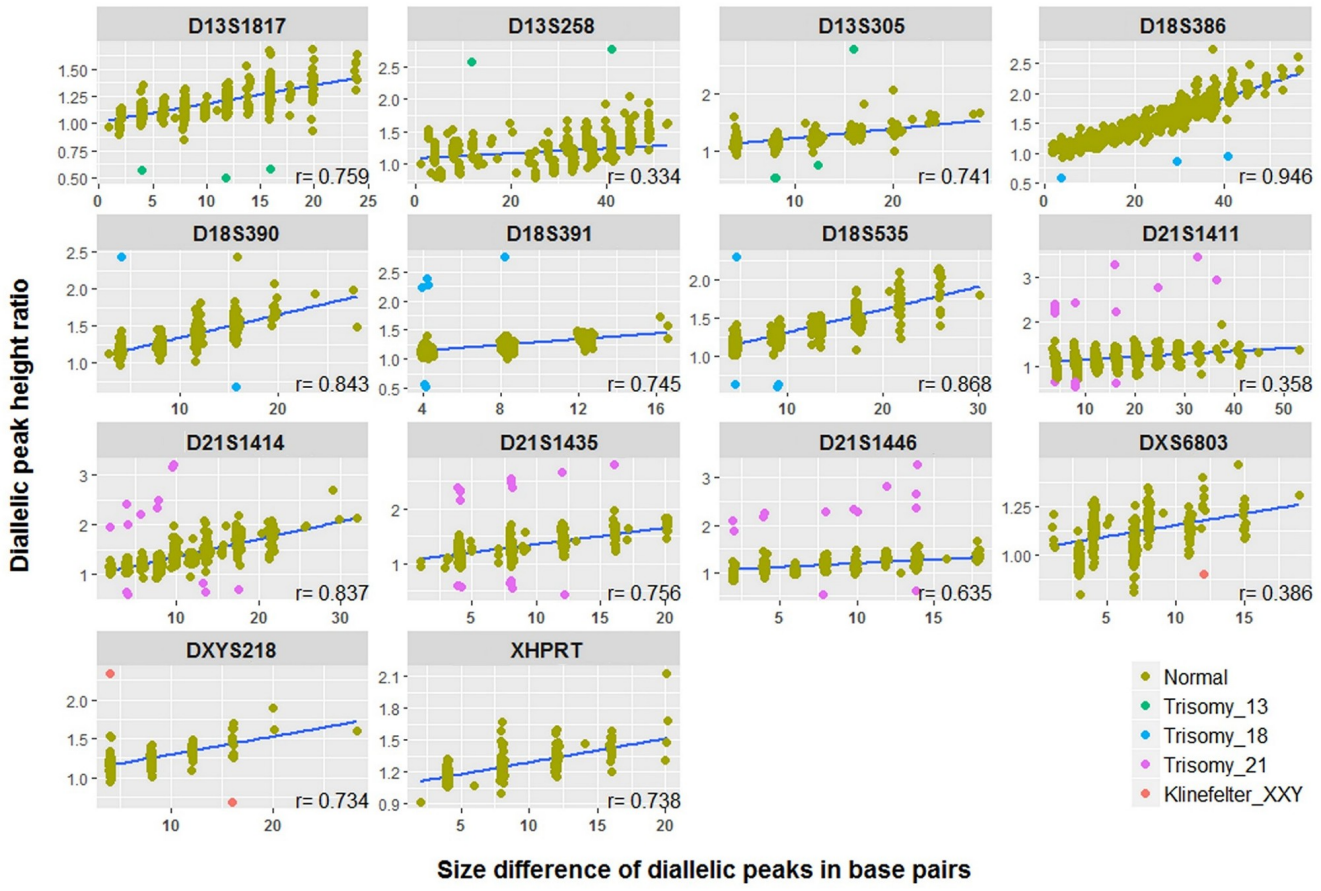
Graphical presentation of the interdependence between the height ratios and the size differences for the diallelic STR markers, given separately for each marker. Correlation coefficients (r) and regression lines for each marker were calculated from normal 1:1 genotypes, while trisomic diallelic genotypes are presented for illustrative purposes only. All samples were run on the ABI 3500 Genetic Analyzer and analyzed with the GeneMapper 4.1 software. The threshold for a minimum peak height detection for each dye was set to ‘factory defaults’ of 50 relative fluorescence units (RFU). The number of height ratios by marker is given in S2 Fig.

When comparing interdependence between height ratios and size difference in STR markers with diallelic pattern, we observed a positive correlation, but the strength of the correlation was variable and ranged from 0.334 to 0.946 (Fig 4). This clearly shows that the height ratio of diallelic STR markers is influenced not only by the base pair size difference of the alleles, but also by a given marker. One way of addressing this variability is by using multilevel regression modeling which accounts for the possible influence of the grouping variables on the final outcome.

The results obtained for the estimated coefficients for both models are presented in Table 1. Although p-values for fixed effects were below a significance level (except for size difference in 1:2 modeling), we think that they should be interpreted with caution because we observed multicollinearity between fixed effects that can lead to inflated standard errors interfering with the inference [37]. However, the presence of multicollinearity will not affect the ability of the model to predict [37,38] especially if the newly predicted data follow the same pattern of multicollinearity [39]. Additionally, due to the uncertainty of the standard errors and non-normal distribution of the fixed effects, we have not used the classical Wald methodology for estimation of the confidence interval of coefficients, but instead we used bootstrapping with 1000 iterations.

**Table 1:** Estimated coefficients and 95% confidence intervals of fixed and random effects obtained by multilevel regression modeling of the 2:1 and 1:2 diallelic ratios.

	Term	Coefficient estimate	Lower 95% CI	Upper 95% CI	P values
**Modeling** **2:1 ratios**	(Intercept)|Marker	130.69	130.31	159.92	
	(Intercept)	-369.66	-375.96	-357.27	<0.0001
	Height Ratio	221.46	193.75	222.08	<0.0001
	Size Difference	-6.78	-7.26	-5.71	<0.0001
**Modeling** **1:2 ratios**	(Intercept)|Marker	111.35	111.28	113.23	
	(Intercept)	326.14	324.84	326.29	<0.0001
	Height Ratio	-427.74	-427.90	-427.56	<0.0001
	Size Difference	0.44	-0.01	0.64	0.083

In order to investigate the relevance of the proposed model, we employed two different approaches. First, for both diallelic modeling approaches (2:1 and 1:2) we fitted three additional models in which we excluded one or both fixed effects from the model and compared the metrics for model selection based on information criteria (Akaike information criterion—AIC, Bayesian information criterion—BIC) [40]. Results showed that the full models including all three parameters were able to better fit the trained data compared to other reduced models (Table 2).

**Table 2:** Results for metrics from an information criteria based analysis for four different models. The lower value means a better fit. Log likelihood (logLik) and deviance are metrics used for computing the AIC.

		logLik	deviance	AIC	BIC	df.residual
**Modeling** **2:1 ratios**	Full model	-6.11	0.02	20.22	48.67	9059
	Model without Size Difference	-36.16	44.68	78.31	99.65	9060
	Model without Height Ratio	-285.84	541.85	577.68	599.02	9060
	Marker only Model	-285.84	541.84	575.69	589.91	9061
**Modeling** **1:2 ratios**	Full model	-7.13	0.02	22.26	50.70	9052
	Model without Size Difference	-10.72	4.00	27.44	48.77	9053
	Model without Height Ratio	-260.16	504.70	526.31	547.64	9053
	Marker only model	-260.16	504.67	524.31	538.53	9054

**Fig 5:**
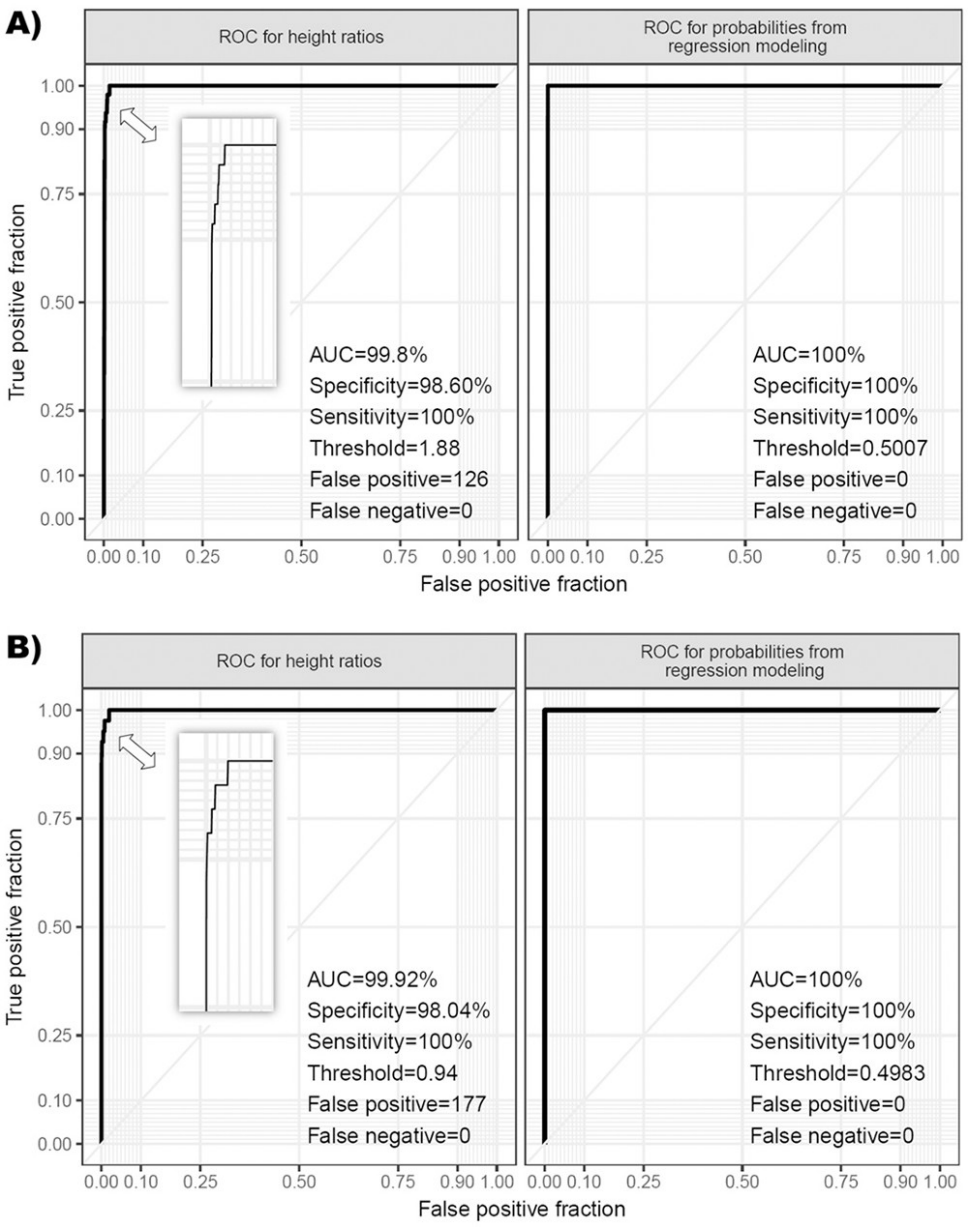
ROC curve analysis comparing prediction accuracy of regression modeling analysis versus the height ratios only analysis. A) Comparison for 2:1 modeling approach and B) Comparison for 1:2 modeling approach.

Secondly, we used a receiver operating characteristic (ROC) curve analysis in order to compare the discrimination ability of the fitted in models versus using the height ratio only for classification of the aneuploidy presence/absence. We converted the fitted values (logits) from the multilevel regression modeling to probabilities for each subject in training data using the formula “probability = exp(logit)/(1+exp(logit))” and then we used those probabilities in the ROC curve analysis for comparison with the height ratio only ROC curve analysis. The results showed that for both modeling approaches the regression analysis outperforms the current approach of using height ratio only for aneuploidy classification of diallelic STR markers (Fig 5A and 5B). In the case of 2:1 height ratio the specificity was 98.6% with 126 false positive cases and in the case of 1:2 height ratio the specificity was 98.04% with 177 false positive cases, but with the regression modeling the specificity was 100% with zero false positives. We would like to emphasize the fact that the values for the thresholds of the "ROC for probabilities from regression modeling" analysis (Fig 5A and 5B) are in fact the mean values between the lowest probability observed for trisomy and the highest probability observed for a normal result in the fitted training data (0.9981 and 0.0034 for 2:1 modeling and 0.9938 and 0.0022 for 1:2 modeling respectively).

When using estimated coefficients and intercepts from the regression modeling, in order to calculate the probability for the classification of a given STR maker as aneuploidic, results from the test cohort of 670 samples showed 100% specificity and sensitivity (S4 Table). When using only the height ratio for classification considering the previously established threshold of 1.94 for markers with 2:1 height ratio and 0.94 for markers with 1:2 ratio, we have observed 82 and 114 false positives, respectively.

In the S4 Table we are presenting the estimated intercepts and coefficients for each marker individually and example calculations as well, for both models. Additionally, here we present an illustrative example of the application of the STR classification using the estimated coefficient from the regression modeling (S6 Fig). It is evident that there is a difference in height ratios when given marker is amplified in multiplex vs. singleton PCR reaction and this variation could be assessed with regression analysis (S6 Fig).

### Treatment of maternal cell contamination in amniotic fluids

The most recent 84 amniocenteses referred to our lab with visible MCC were treated with the simple physical separation of potentially contaminating material and, as described, for each sample a separate isolation and QF-PCR analysis was performed for both supernatant and precipitate of which, 38 showed identical profile for both isolations which was different from the profile obtained from the mother. This means that the potentially contaminating blood in fact originated from the fetus, and a conclusive finding could be issued. Of the remaining 46 samples, 35 showed two profiles (from the mother and fetus) for the isolation from the precipitate and one profile (from the fetus) for the isolation from the supernatant, thus a conclusive finding was issued for these samples, as well. The remaining 11 samples showed two profiles (from the mother and fetus) in both isolations, and the final status of the chromosomes for these samples remained unresolved. This gives us a rate of 76% of successful reporting the truly MCC amniotic fluids.

## Discussion

Taking into account the wide application, not only in prenatal diagnostics but also for other purposes, it is reasonable to address some technical deficiencies of QF-PCR and try to make some improvements. In our study, we tried to assess and improve the reliability of the classification of diallelic STR markers, used for the determination of aneuploidies, which currently is based on their height ratio. External factors that can influence the reproducibility of the height ratio of the diallelic peaks and the QF-PCR reaction in general are the low quality of the DNA and the large number of co-amplified markers in a single multiplex reaction. Isolated DNA of fetal origin is usually fragmented to varying degrees and is of poorer quality relative to DNA isolated from blood or tissue, and such fragmentation is likely to be the cause of variability in the PCR reaction. In order to improve the robustness of the QF-PCR analysis, we combined 20 different primer pairs in a single reaction, targeting 26 positions in the genome. If the potential heterozygosity of the STR markers is taken into account, then the reaction can result in amplification of up to 37 PCR fragments in male fetuses and 32 in female fetuses. Such simultaneous amplification of a large and variable number of PCR fragments can also affect reproducibility. However, since the aforementioned factors cannot be influenced, other factors could be used to ameliorate the QF-PCR analysis. Using the height ratio and size difference of the diallelic STR peaks as fixed effects in the regression modelling, while at the same time taking into account the variability each marker has on the relationship between them, allows more accurate classification of the STR markers as compared to using only the height ratios. The primary purpose of the proposed method of classification is to improve the reliability of the analysis and to eliminate the need for re-testing due to the presence of an indecisive value for "normal" height ratio. We would like to emphasize that even with this way of classifying, the possibility of a presence of a single nucleotide polymorphism in the target region of the primers must not be neglected; thus the final results should be always based on the presence of two or more heterozygous markers for a particular chromosome.

Considering the relatively large number of analyzed samples for prenatal diagnostics, we also present diagnostic data and data related to marker mutations within the study. We noticed differences in the distribution of the proportions of aggregated individual observations of maternal age and gestational age between different types of aneuploidies. For the maternal age, the observed differences were in agreement with previously observed statistically significant correlation between increased age and trisomies 21 and 18 particularly [41]. For gestational week at diagnosis the difference between trisomy 21 and other trisomies is probably due to the relationship between earlier detection and the severity of the symptoms of the different types of aneuploidies. Regarding the markers, it is noticeable that we observed a high frequency of duplications for the pseudoautosomal DYS218 marker, which is also reported by others [42]. To eliminate the presence of pathogenic duplication on the X chromosome in the region of DYS218 and to confirm a benign duplication of this marker in one of the parents, we always analyzed both parents. An additional marker in the close proximity of the DYS218 can be used as a substitute.

Designing a one-tube QF-PCR multiplex reaction does not eliminate the need for performing additional, chromosome specific QF-PCR reactions. The number of STR markers used per chromosome and heterozygosity of the markers used are the factors that should be taken into account.

Except for the QF-PCR reaction itself, in this study we present our experience in treating amniotic fluids contaminated with maternal material. Such treatment has enabled us to successfully eliminate the maternal contamination in 76% of the cases. We believe that the treatment for visible maternal cell contamination is beneficial for patients. The usefulness could be significant not only for diagnosing common aneuploidies but also in cases of prenatal diagnosis of other genetic disorders (for example prenatal diagnosis of monogenic diseases). A similar procedure has already been published in the literature for the extraction of cffDNA with commercial kits and its use for QF-PCR and/or aCGH analyses [43,44]. Our methodology consists of spontaneous precipitation, allowing physical separation of the contaminated material. In this way, a similar effect is achieved with our procedure as with the classical cytogenetic analysis in which the cultivation of cells favours the development of amniocytes and reduces or eliminates the material from the mother [45,46]. The advantage of the presented treatment of the amniotic fluids with MCC is that it does not require a complicated preparation of the samples before their analysis. The negative side of this type of treatment is that the duration of the analysis is being extended for approximately one day, but taking into account that when using the proposed treatment the probability of issuing a conclusive result for invasive procedure such as amniocentesis increases significantly, seems like a reasonable compromise. Similar effect, as precipitation, can be achieved with centrifugation of the amniotic fluid at low speed, but we prefer the precipitation method which, although it takes more time, is more reliable. This is because the degree of contamination cannot be known in advance and it would be difficult to determine the speed and duration of the centrifugation in order to eliminate the supernatant contaminating cells to a satisfactory degree while at the same time retaining a sufficient amount of fetus material to perform the successful QF- PCR analysis.

In conclusion, here we present a one-tube multiplex QF-PCR analysis protocol, including multilevel regression modeling for better interpretation of diallelic STR markers, complemented with appropriate treatment of contaminated amniotic fluids, thus eliminating sample re-testing and reinforcing the robustness of the QF-PCR method for prenatal testing.

## Supporting information

S1 Table Primers used in the QF-PCR reaction. Oligonucleotide sequences, fluorescent dye labeling of forward primers, GC percentage, primer melting temperatures (Tm), size ranges of the alleles, heterozygosityand the genomic positions of the amplified regions are shown. Heterozygosity of the DXS6803 and HPRT markers was calculated only from female samples.
(XLS)

S2 Table Additional primers used as back-up to the main QF-PCR multiplex reaction. Oligonucleotide sequences, fluorescent dye labeling of forward primers, GC percentage, primer melting temperatures (Tm) and the genomic positions of the amplified regions are shown. Primers for chromosomes 13, 18 and 21 were combined as per chromosome multiplex PCR reaction. In each of the mixes for chr 13, 18 and 21 we have included primer pair already present in the main QF-PCR mix which allows confirmation of the identity of the sample. Primers for sex chromosomes were used as a single PCR reactions.
(XLS)

S3 Table Dataset of the cohort of 871 samples used in the regression analysis. Data for all markers and data for heterozygous alleles only is shown in separate worksheets.
(XLSX)

S4 Table Dataset used to test the prediction accuracy of the regression modeling. Example calculations are presented in separate worksheet.
(XLSX)

S1 Fig Chromosomal locations of the QF-PCR markers used as a back-up of the main QF-PCR reaction. Markers are colored by the chromosome/chromosomes they are designed to target.
(PDF)

S2 Fig Graphical presentation of the number of the height ratios used in multilevel regression analysis, given by marker. For each marker the number of Normal 1:1 (black color), Trisomic 2:1 (red color) and Trisomic 1:2 (blue color) height ratios is presented.
(PDF)

S3 Fig Illustration of the treatment of the contaminated amniotic fluids. Contaminated amniotic fluid is precipitated for 6–12 hours and then **A)** Upper clear phase (green color) was collected resulting in amplification of a pure uncontaminated profile of the fetus and **B)** Afterwards, lower contaminated phase (blue color) was collected separately wherein the amplification showed maternal cell contamination. Blue circles show the contaminating alleles from the mother.
(PDF)

S4 Fig Detailed distribution of the counts of the maternal age by different types of aneuploidies. Size of the circles corresponds to the number of patients in the given age group. In addition, they are colored differently for better visualization.
(PDF)

S5 Fig Detailed distribution of the counts of the maternal gestational age by different types of aneuploidies. Size of the circles corresponds to the number of patients in the given gestational age group. In addition, they are colored differently for better visualization.
(PDF)

S6 Fig Illustrative example of the application of the STR classification using the estimated coefficient from regression modeling. A) Electropherogram of multiplex QF-PCR from healthy pregnant woman. Two STR’s (D18S390 and D18S535) have been marked because they have height ratio of 2.05 and 2.01, respectively, thus indicative for the presence of aneuploidy. Calculations with coefficients obtained with regression modeling gives low probability for aneuploidy. B) For comparison, electropherograms from single PCR reactions of the two STR’s from the same DNA sample are given. Height ratios are within normal ranges in singleton PCR reactions, concordant with the multiplex QF-PCR/regression analysis results.
(PDF)

## References

[1] Carlson LM, Vora NL Prenatal Diagnosis: Screening and Diagnostic Tools. Obstet Gynecol Clin North Am. 2017; 44: 245–256. 10.1016/j.ogc.2017.02.004 28499534 PMC5548328

[2] Shaffer LG, Bui TH Molecular cytogenetic and rapid aneuploidy detection methods in prenatal diagnosis. Am J Med Genet C Semin Med Genet. 2007; 145C: 87–98. 10.1002/ajmg.c.30114 17290441

[3] Hahn S, Jackson LG, Zimmermann BG Prenatal diagnosis of fetal aneuploidies: post-genomic developments. Genome Med. 2010; 2: 50 10.1186/gm171 20687900 PMC2945007

[4] Fan HC, Blumenfeld YJ, Chitkara U, Hudgins L, Quake SR Noninvasive diagnosis of fetal aneuploidy by shotgun sequencing DNA from maternal blood. Proc Natl Acad Sci U S A. 2008; 105: 16266–16271. 10.1073/pnas.0808319105 18838674 PMC2562413

[5] Pertl B, Yau SC, Sherlock J, Davies AF, Mathew CG, Adinolfi M Rapid molecular method for prenatal detection of Down’s syndrome. Lancet. 1994; 343: 1197–1198. 10.1016/s0140-6736(94)92404-x 7909872

[6] Mansfield ES Diagnosis of Down syndrome and other aneuploidies using quantitative polymerase chain reaction and small tandem repeat polymorphisms. Hum Mol Genet. 1993; 2: 43–50. 10.1093/hmg/2.1.43 8490622

[7] Cirigliano V, Voglino G, Ordonez E, Marongiu A, Paz Canadas M, Ejarque M, et al Rapid prenatal diagnosis of common chromosome aneuploidies by QF-PCR, results of 9 years of clinical experience. Prenat Diagn. 2009; 29: 40–49. 10.1002/pd.2192 19173345

[8] Mann K, Hills A, Donaghue C, Thomas H, Ogilvie CM Quantitative fluorescence PCR analysis of >40,000 prenatal samples for the rapid diagnosis of trisomies 13, 18 and 21 and monosomy X. Prenat Diagn. 2012; 32: 1197–1204. 10.1002/pd.3986 23097180

[9] Speevak MD, McGowan-Jordan J, Chun K The detection of chromosome anomalies by QF-PCR and residual risks as compared to G-banded analysis. Prenat Diagn. 2011; 31: 454–458. 10.1002/pd.2716 21500231

[10] Langlois S, Duncan A Use of a DNA method, QF-PCR, in the prenatal diagnosis of fetal aneuploidies. J Obstet Gynaecol Can. 2011; 33: 955–960. 10.1016/s1701-2163(16)35022-8 21923994

[11] Kozlowski P, Grund I, Hickmann G, Stressig R, Knippel AJ Quantitative fluorescent polymerase chain reaction versus cytogenetics: risk-related indication and clinical implication of nondetected chromosomal disorders. Fetal Diagn Ther. 2006; 21: 217–223. 10.1159/000089306 16491006

[12] Van Opstal D, Srebniak MI Cytogenetic confirmation of a positive NIPT result: evidence-based choice between chorionic villus sampling and amniocentesis depending on chromosome aberration. Expert Rev Mol Diagn. 2016; 16: 513–520. 10.1586/14737159.2016.1152890 26864482

[13] Dey M, Sharma S, Aggarwal S Prenatal screening methods for aneuploidies. N Am J Med Sci. 2013; 5: 182–190. 10.4103/1947-2714.109180 23626953 PMC3632021

[14] Pan M, Li FT, Li Y, Jiang FM, Li DZ, Lau TK, et al Discordant results between fetal karyotyping and non-invasive prenatal testing by maternal plasma sequencing in a case of uniparental disomy 21 due to trisomic rescue. Prenat Diagn. 2013; 33: 598–601. 10.1002/pd.4069 23533085

[15] Plaseski T, Noveski P, Trivodalieva S, Efremov GD, Plaseska-Karanfilska D Quantitative fluorescent-PCR detection of sex chromosome aneuploidies and AZF deletions/duplications. Genet Test. 2008; 12: 595–605. 10.1089/gte.2008.0068 19072570

[16] Plaseska-Karanfilska D, Noveski P, Plaseski T (2011) Detection of the most common genetic causes of male infertility by quantitative fluorescent (QF)-PCR analysis. Human Genetic Diseases: InTech.

[17] Noveski P, Madjunkova S, Sukarova Stefanovska E, Matevska Geshkovska N, Kuzmanovska M, Dimovski A, et al Loss of Y Chromosome in Peripheral Blood of Colorectal and Prostate Cancer Patients. PLoS One. 2016; 11: e0146264 10.1371/journal.pone.0146264 26745889 PMC4706411

[18] Kimura A, Hishimoto A, Otsuka I, Okazaki S, Boku S, Horai T, et al Loss of chromosome Y in blood, but not in brain, of suicide completers. PLoS One. 2018; 13: e0190667 10.1371/journal.pone.0190667 29300758 PMC5754120

[19] Dumanski JP, Lambert JC, Rasi C, Giedraitis V, Davies H, Grenier-Boley B, et al Mosaic Loss of Chromosome Y in Blood Is Associated with Alzheimer Disease. Am J Hum Genet. 2016; 98: 1208–1219. 10.1016/j.ajhg.2016.05.014 27231129 PMC4908225

[20] Haitjema S, Kofink D, van Setten J, van der Laan SW, Schoneveld AH, Eales J, et al Loss of Y Chromosome in Blood Is Associated With Major Cardiovascular Events During Follow-Up in Men After Carotid Endarterectomy. Circ Cardiovasc Genet. 2017; 10: e001544 10.1161/circgenetics.116.001544 28768751

[21] Forsberg LA, Rasi C, Malmqvist N, Davies H, Pasupulati S, Pakalapati G, et al Mosaic loss of chromosome Y in peripheral blood is associated with shorter survival and higher risk of cancer. Nat Genet. 2014; 46: 624–628. 10.1038/ng.2966 24777449 PMC5536222

[22] Stojilkovic-Mikic T, Mann K, Docherty Z, Mackie Ogilvie C Maternal cell contamination of prenatal samples assessed by QF-PCR genotyping. Prenat Diagn. 2005; 25: 79–83. 10.1002/pd.1089 15662689

[23] Nicolini U, Lalatta F, Natacci F, Curcio C, Bui TH The introduction of QF-PCR in prenatal diagnosis of fetal aneuploidies: time for reconsideration. Hum Reprod Update. 2004; 10: 541–548. 10.1093/humupd/dmh046 15514017

[24] Cirigliano V, Ejarque M, Canadas MP, Lloveras E, Plaja A, Perez MM, et al Clinical application of multiplex quantitative fluorescent polymerase chain reaction (QF-PCR) for the rapid prenatal detection of common chromosome aneuploidies. Mol Hum Reprod. 2001; 7: 1001–1006. 10.1093/molehr/7.10.1001 11574670

[25] Hulten MA, Dhanjal S, Pertl B Rapid and simple prenatal diagnosis of common chromosome disorders: advantages and disadvantages of the molecular methods FISH and QF-PCR. Reproduction. 2003; 126: 279–297. 10.1530/rep.0.1260279 12968936

[26] Pertl B, Kopp S, Kroisel PM, Hausler M, Sherlock J, Winter R, et al Quantitative fluorescence polymerase chain reaction for the rapid prenatal detection of common aneuploidies and fetal sex. Am J Obstet Gynecol. 1997; 177: 899–906. 10.1016/s0002-9378(97)70292-8 9369843

[27] Hastings R, Howell R, Bricarelli F, Kristoffersson U, Cavani S A common European framework for quality assessment for constitutional, acquired and molecular cytogenetic investigations. Eur Cytogenet Assoc Newsl. 2012; 30: 11–19.

[28] Noveski P, Terzic M, Vujovic M, Kuzmanovska M, Stefanovska ES, Plaseska-Karanfilska D Quantitative fluorescent polymerase chain reaction (QF-PCR) for the rapid prenatal diagnosis of common fetal aneuploidies. protocols.io 10.17504/protocols.io.2v9ge96

[29] Sherlock J, Cirigliano V, Petrou M, Tutschek B, Adinolfi M Assessment of diagnostic quantitative fluorescent multiplex polymerase chain reaction assays performed on single cells. Ann Hum Genet. 1998; 62: 9–23. 10.1046/j.1469-1809.1998.6210009.x 9659974

[30] Cirigliano V, Voglino G, Canadas MP, Marongiu A, Ejarque M, Ordonez E, et al Rapid prenatal diagnosis of common chromosome aneuploidies by QF-PCR. Assessment on 18,000 consecutive clinical samples. Mol Hum Reprod. 2004; 10: 839–846. 10.1093/molehr/gah108 15361554

[31] R Core Team (2017) R: a language and environment for statistical computing. Vienna, Austria: R Foundation for Statistical Computing; 2017.

[32] Wickham H Tidyverse: Easily install and load’tidyverse’packages. R package version. 2017; 1:

[33] Bates D, Maechler M, Bolker B, Walker S (2015) lme4: Linear mixed-effects models using Eigen and S4. R package version 1.1–7. 2014.

[34] Robin X, Turck N, Hainard A, Tiberti N, Lisacek F, Sanchez JC, et al pROC: an open-source package for R and S+ to analyze and compare ROC curves. BMC Bioinformatics. 2011; 12: 77 10.1186/1471-2105-12-77 21414208 PMC3068975

[35] Sachs MC plotROC: A Tool for Plotting ROC Curves. Journal of Statistical Software. 2017; 79:10.18637/jss.v079.c02 PMC6347406 30686944

[36] Wolfe D, Dudek S, Ritchie MD, Pendergrass SA Visualizing genomic information across chromosomes with PhenoGram. BioData Min. 2013; 6: 18 10.1186/1756-0381-6-18 24131735 PMC4015356

[37] Shmueli G To explain or to predict? Statistical science. 2010: 289–310.

[38] Vaughan TS, Berry KE Using Monte Carlo techniques to demonstrate the meaning and implications of multicollinearity. Journal of Statistics Education. 2005; 13:

[39] Gujarati D, Porter D Multicollinearity: What happens if the regressors are correlated. Basic econometrics. 2003; 363:

[40] Aho K, Derryberry D, Peterson T Model selection for ecologists: the worldviews of AIC and BIC. Ecology. 2014; 95: 631–636. 24804445 10.1890/13-1452.1

[41] Kim YJ, Lee JE, Kim SH, Shim SS, Cha DH Maternal age-specific rates of fetal chromosomal abnormalities in Korean pregnant women of advanced maternal age. Obstet Gynecol Sci. 2013; 56: 160–166. 10.5468/ogs.2013.56.3.160 24327996 PMC3784117

[42] Badenas C, Rodriguez-Revenga L, Morales C, Mediano C, Plaja A, Perez-Iribarne MM, et al Assessment of QF-PCR as the first approach in prenatal diagnosis. J Mol Diagn. 2010; 12: 828–834. 10.2353/jmoldx.2010.090224 20889556 PMC2963915

[43] Madjunkova S, Tong Li C, Vlasschaert M, Adams M, Chitayat D, Maire G, et al QF-PCR rapid aneuploidy screen and aCGH analysis of cell free fetal (cff) DNA in supernatant of compromised amniotic fluids (AF). Prenat Diagn. 2014; 34: 970–976. 10.1002/pd.4405 24801814

[44] Lapaire O, Lu XY, Johnson KL, Jarrah Z, Stroh H, Cowan JM, et al Array-CGH analysis of cell-free fetal DNA in 10 mL of amniotic fluid supernatant. Prenat Diagn. 2007; 27: 616–621. 10.1002/pd.1752 17510923

[45] Nagan N, Faulkner NE, Curtis C, Schrijver I Laboratory guidelines for detection, interpretation, and reporting of maternal cell contamination in prenatal analyses a report of the association for molecular pathology. J Mol Diagn. 2011; 13: 7–11. 10.1016/j.jmoldx.2010.11.013 21227389 PMC3069929

[46] Winsor EJ, Silver MP, Theve R, Wright M, Ward BE Maternal cell contamination in uncultured amniotic fluid. Prenat Diagn. 1996; 16: 49–54. 10.1002/(sici)1097-0223(199601)16:1<49::aid-pd808>3.0.co;2-u 8821852

