# Supplementary material for: Multilevel regression modeling for aneuploidy classification and physical separation of maternal cell contamination facilitates the QF-PCR based analysis of common fetal aneuploidies
Source: PLoS One. 2019 Aug 20;14(8):e0221227. doi: 10.1371/journal.pone.0221227 (PMC6701765; doi:10.1371/journal.pone.0221227)
Supplement: S1 Fig — Markers are colored by the chromosome/chromosomes they are designed to target. (PDF) [file pone.0221227.s005.pdf]

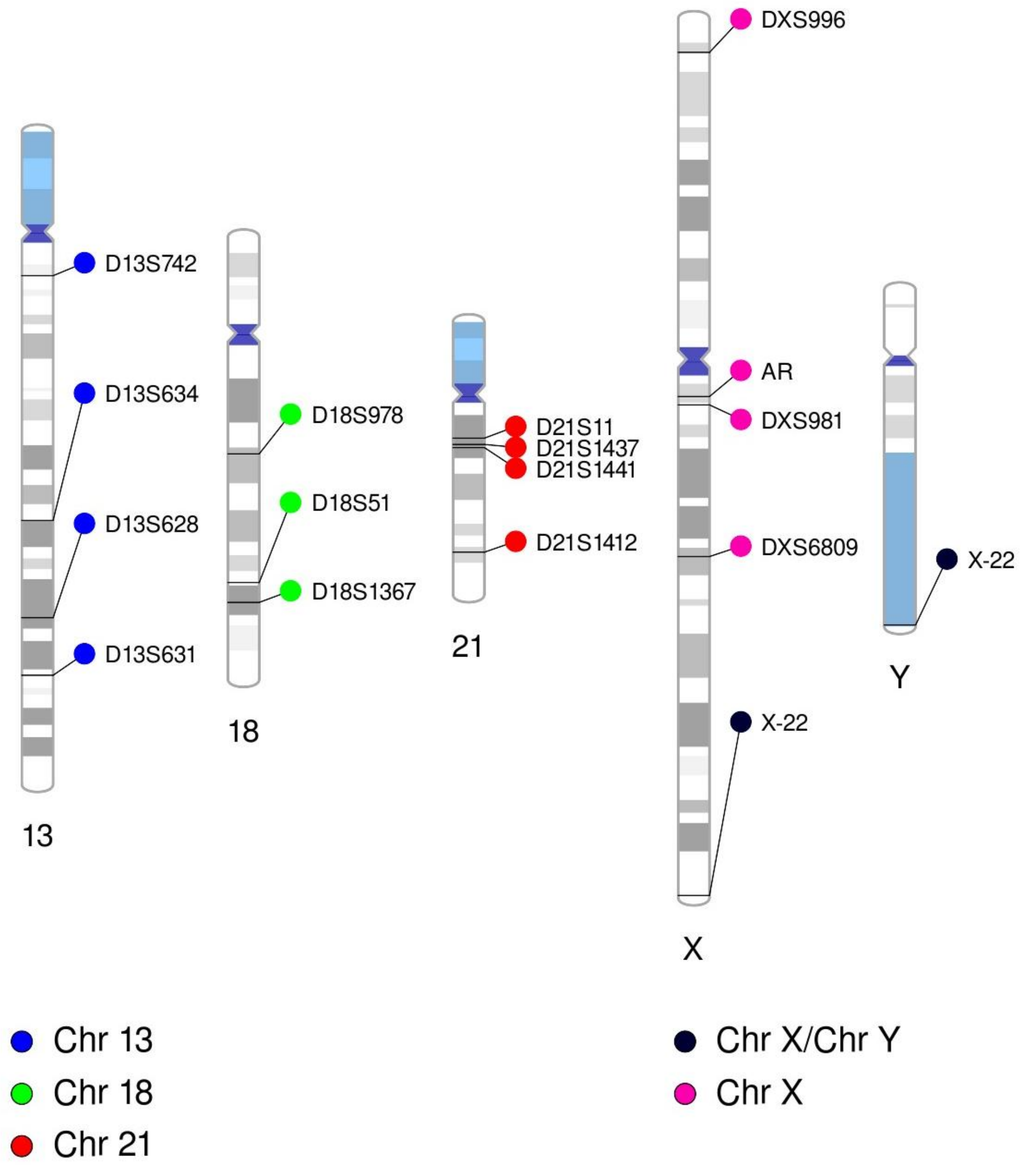

<u>S1 Fig.</u> Chromosomal locations of the QF-PCR markers used as a back-up of the main QF-PCR reaction. Markers are colored by the chromosome/chromosomes they are designed to target.
