# Supplementary material for: Multilevel regression modeling for aneuploidy classification and physical separation of maternal cell contamination facilitates the QF-PCR based analysis of common fetal aneuploidies
Source: PLoS One. 2019 Aug 20;14(8):e0221227. doi: 10.1371/journal.pone.0221227 (PMC6701765; doi:10.1371/journal.pone.0221227)
Supplement: S2 Fig — For each marker the number of Normal 1:1 (black color), Trisomic 2:1 (red color) and Trisomic 1:2 (blue color) height ratios is presented. (PDF) [file pone.0221227.s006.pdf]

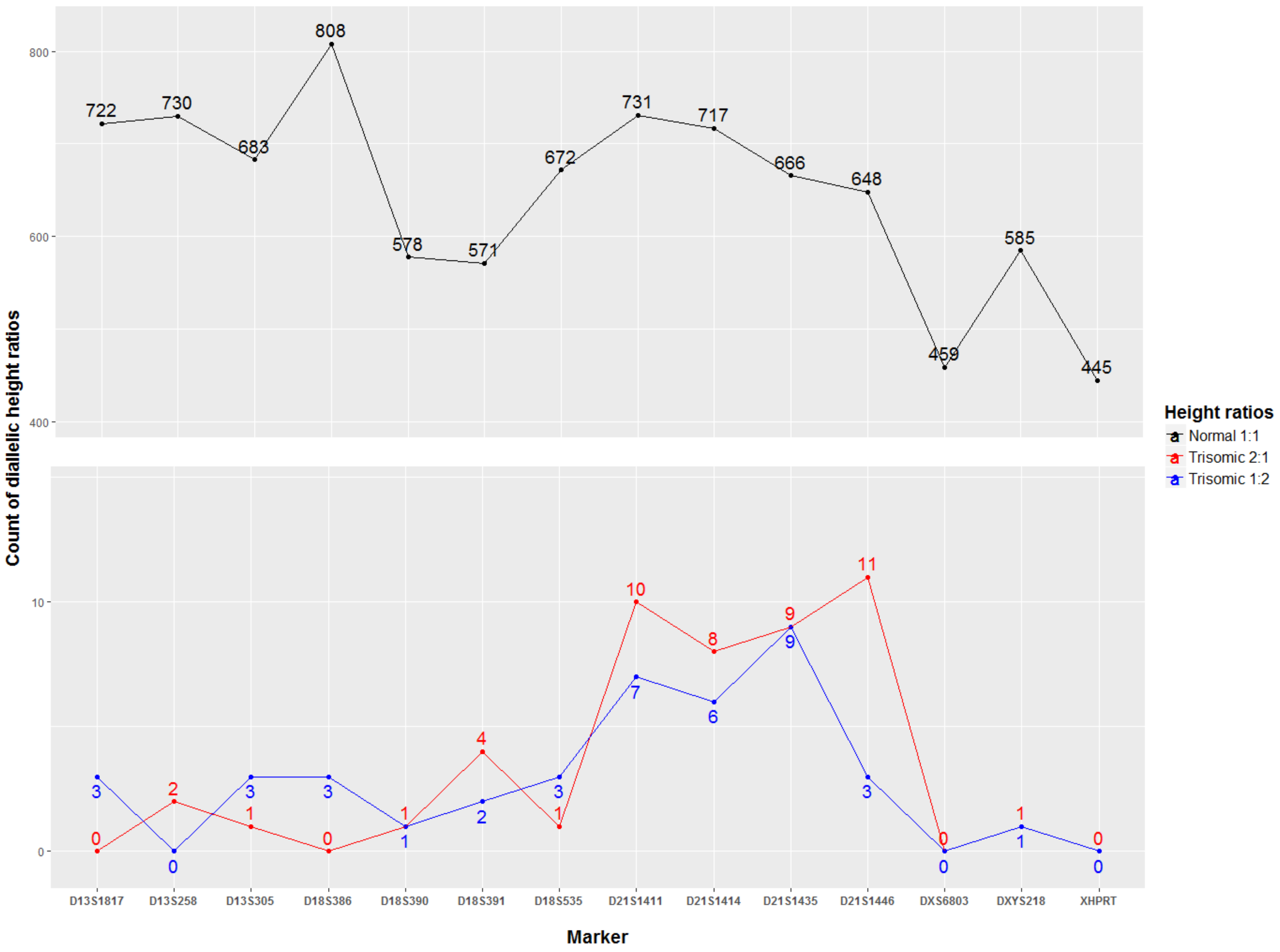


**<u>S2 Fig.</u> Graphical presentation of the number of the height ratios used in multilevel regression analysis, given by marker.** For each marker the number of Normal 1:1 (black color), Trisomic 2:1 (red color) and Trisomic 1:2 (blue color) height ratios is presented.
