# Supplementary material for: Multilevel regression modeling for aneuploidy classification and physical separation of maternal cell contamination facilitates the QF-PCR based analysis of common fetal aneuploidies
Source: PLoS One. 2019 Aug 20;14(8):e0221227. doi: 10.1371/journal.pone.0221227 (PMC6701765; doi:10.1371/journal.pone.0221227)
Supplement: S3 Fig — Contaminated amniotic fluid is precipitated for 6–12 hours and then A) Upper clear phase (green color) was collected resulting in amplification of a pure uncontaminated profile of the fetus and B) Afterwards, lower contaminated phase (blue color) was collected separately wherein the amplification showed maternal cell contamination. Blue circles show the contaminating alleles from the mother. (PDF) [file pone.0221227.s007.pdf]

A)

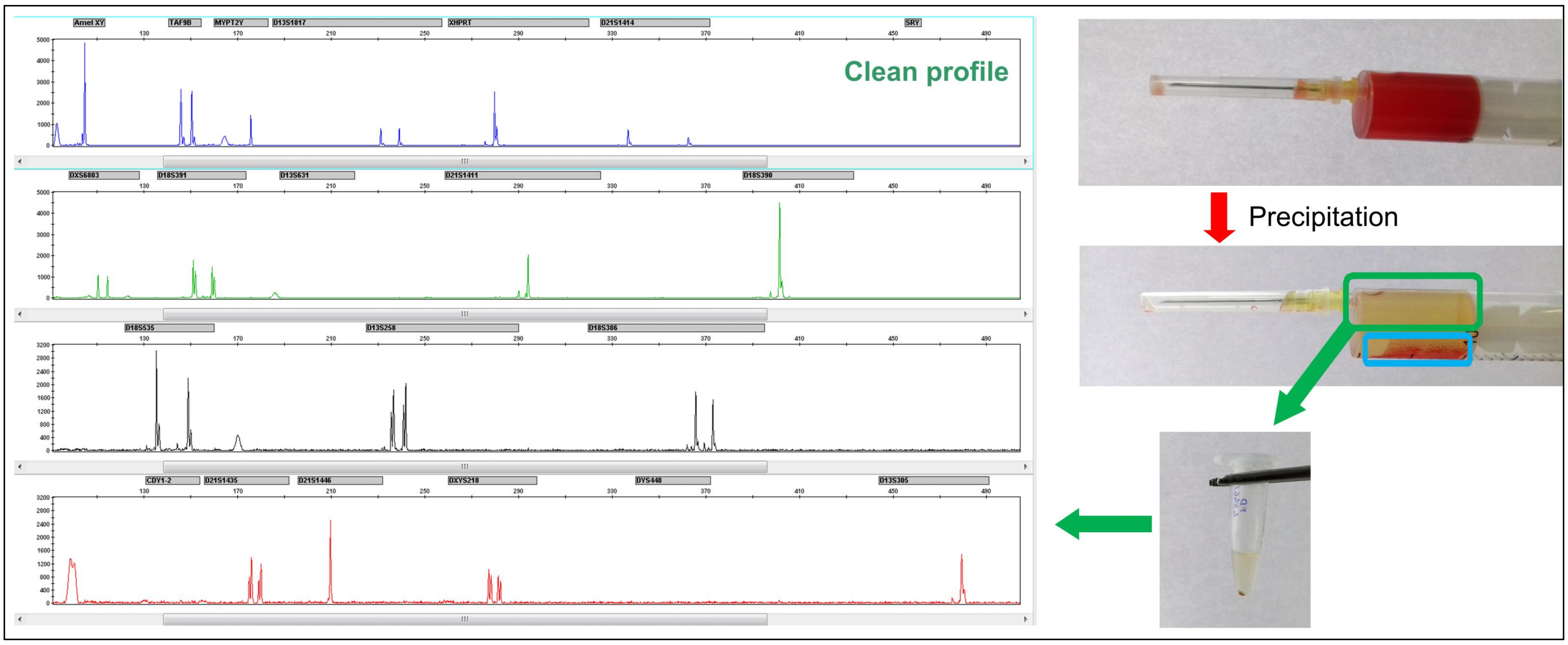

B)

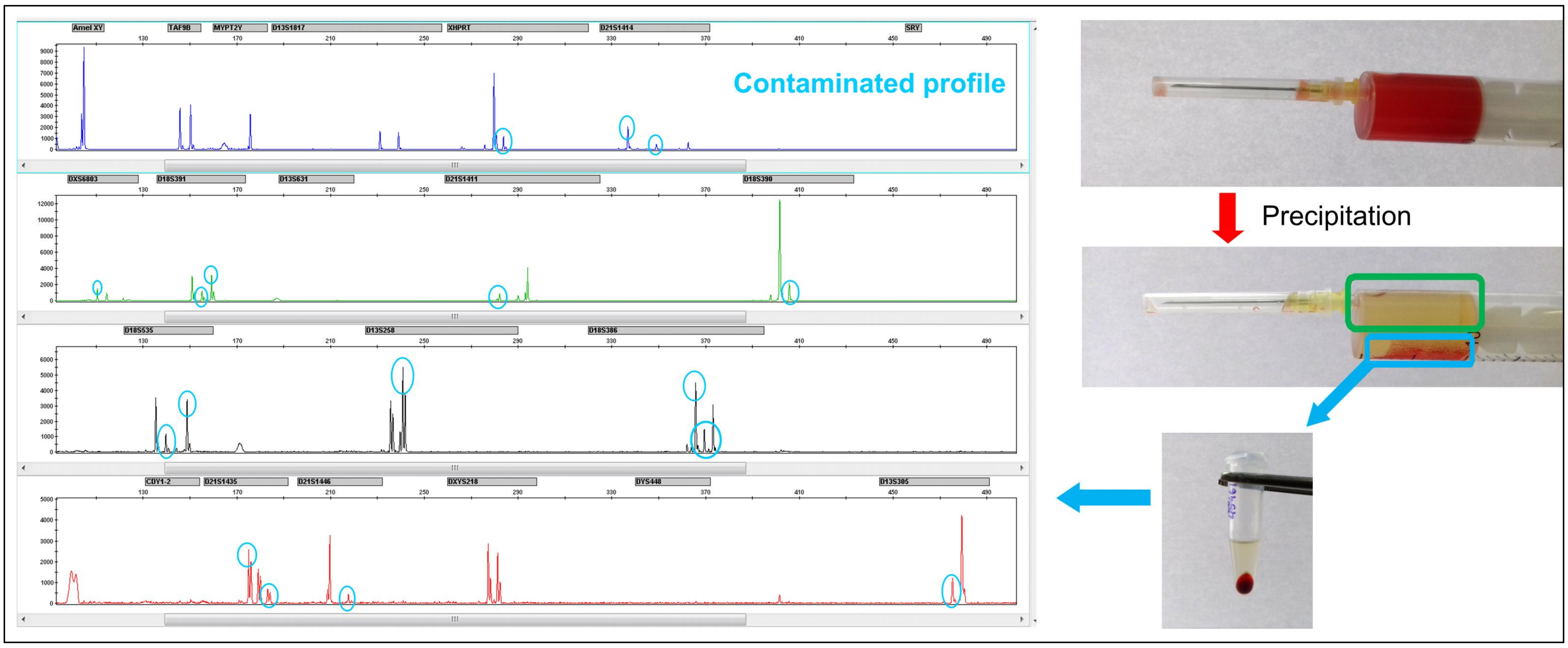

S3 Fig. Illustration of the treatment of the contaminated amniotic fluids. Contaminated amniotic fluid is precipitated for 6-12 hours and then **A)** Upper clear phase (green color) was collected resulting in amplification of a pure uncontaminated profile of the fetus and **B)** Afterwards, lower contaminated phase (blue color) was collected separately wherein the amplification showed maternal cell contamination. Blue circles show the contaminating alleles from the mother.
