# Supplementary material for: Multilevel regression modeling for aneuploidy classification and physical separation of maternal cell contamination facilitates the QF-PCR based analysis of common fetal aneuploidies
Source: PLoS One. 2019 Aug 20;14(8):e0221227. doi: 10.1371/journal.pone.0221227 (PMC6701765; doi:10.1371/journal.pone.0221227)
Supplement: S4 Fig — Size of the circles corresponds to the number of patients in the given age group. In addition, they are colored differently for better visualization. (PDF) [file pone.0221227.s008.pdf]

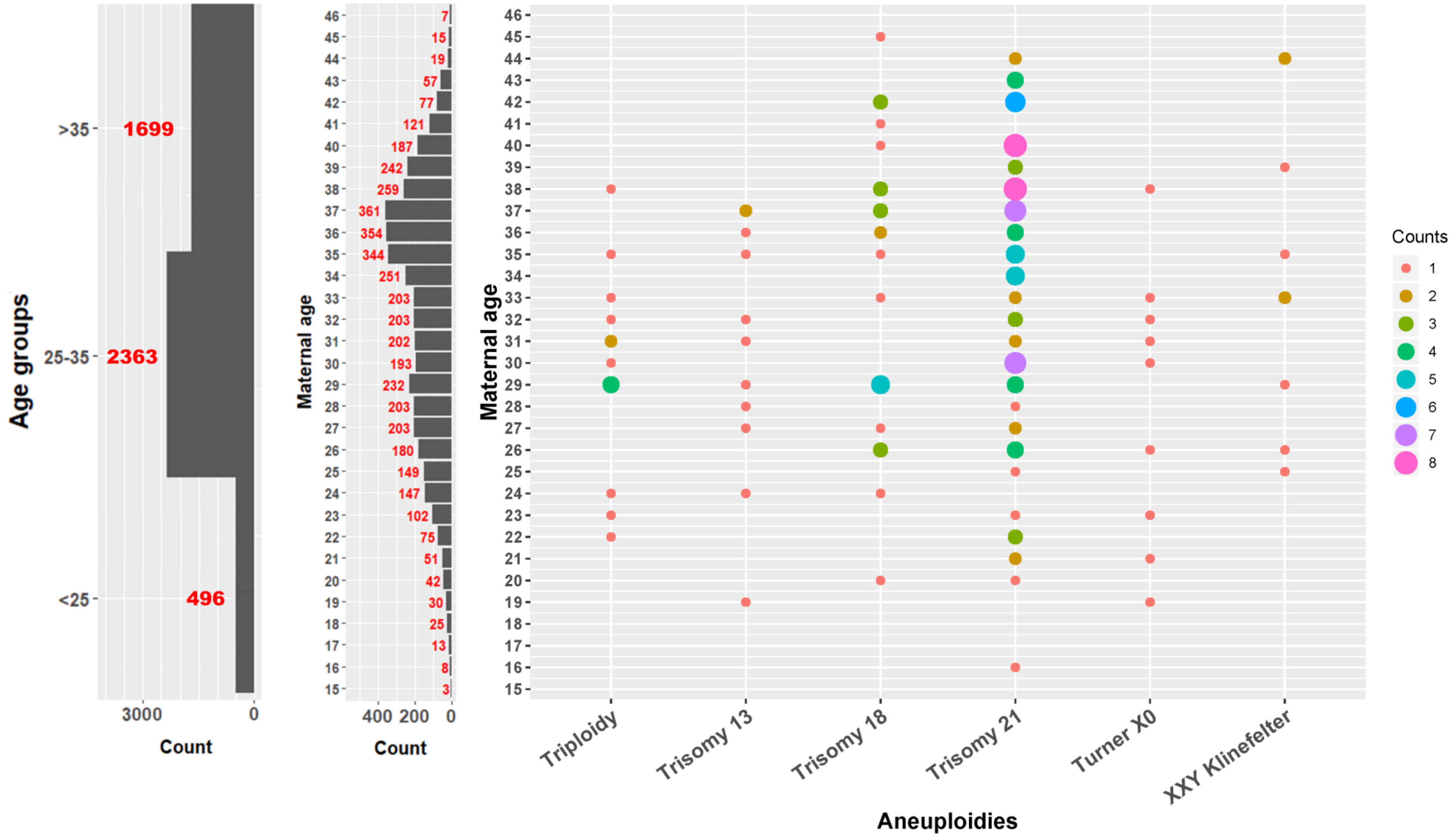

S4 Fig. Detailed distribution of the counts of the maternal age by different types of aneuploidies. Size of the circles corresponds to the number of patients in the given age group. In addition, they are colored differently for better visualization. As numbers of pregnant women is variable by age, we are also presenting the detailed counts of the diagnosed pregnant women by age (n=4558 with available data), and additionally, the counts by age groups according to the groups presented in Fig 3A.
