# Supplementary figures and images for: Multilevel regression modeling for aneuploidy classification and physical separation of maternal cell contamination facilitates the QF-PCR based analysis of common fetal aneuploidies
Source: PLoS One. 2019 Aug 20;14(8):e0221227. doi: 10.1371/journal.pone.0221227 (PMC6701765; doi:10.1371/journal.pone.0221227)

A)

Multiplex PCR

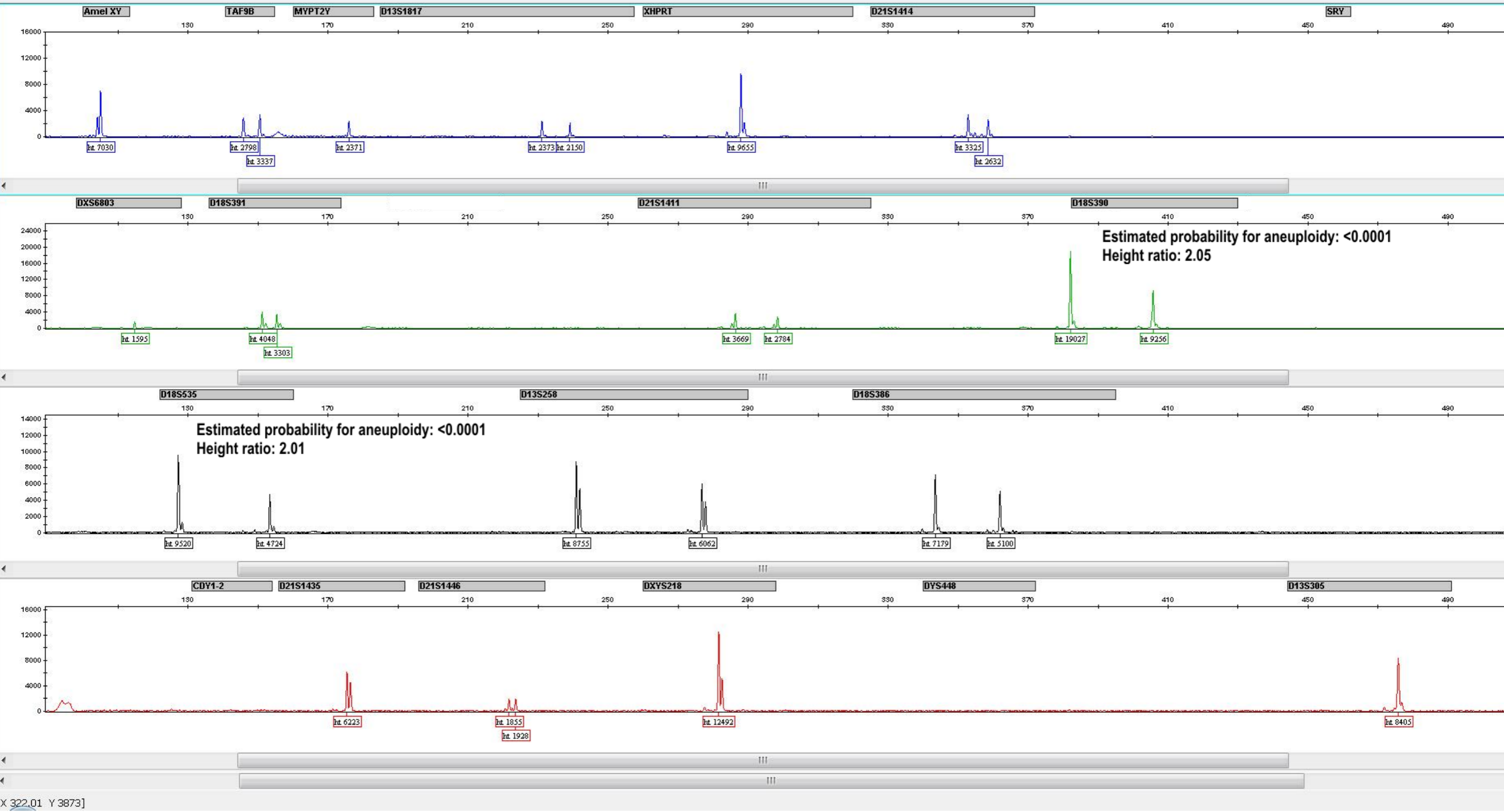

B)

D18S390 - Single PCR

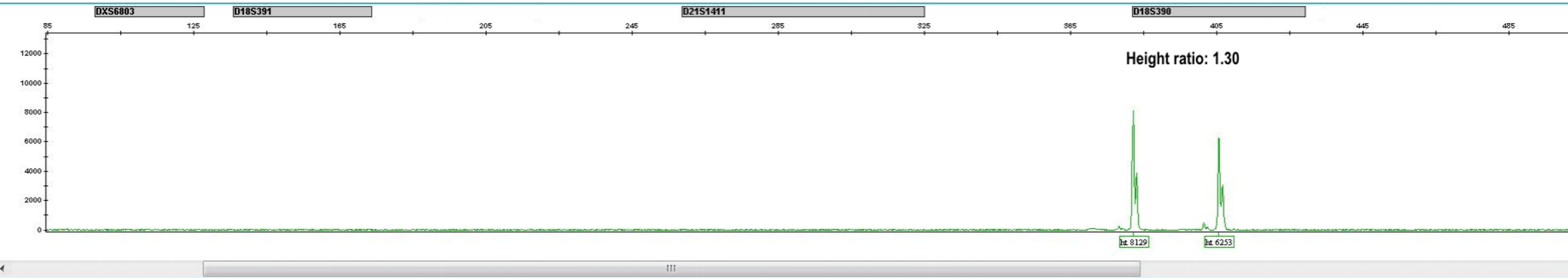

D18S535 - Single PCR

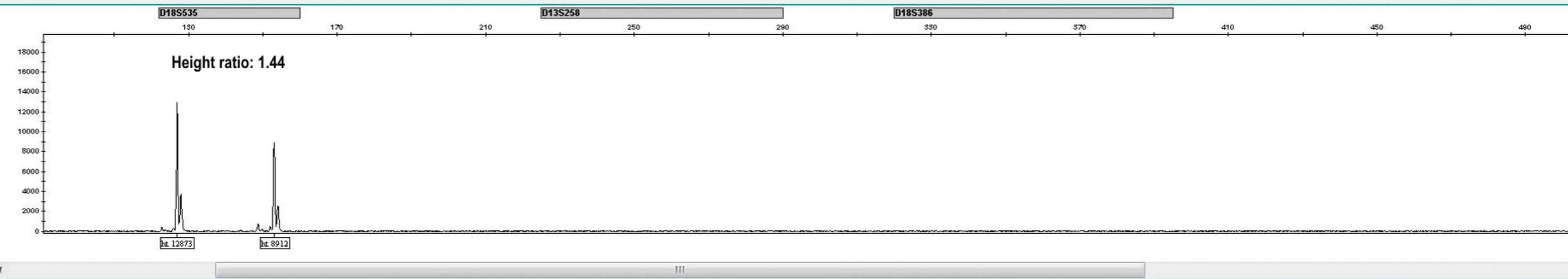

Supplement: S6 Fig — A) Electropherogram of multiplex QF-PCR from healthy pregnant woman. Two STR’s (D18S390 and D18S535) have been marked because they have height ratio of 2.05 and 2.01, respectively, thus indicative for the presence of aneuploidy. Calculations with coefficients obtained with regression modeling gives low probability for aneuploidy. B) For comparison, electropherograms from single PCR reactions of the two STR’s from the same DNA sample are given. Height ratios are within normal ranges in singleton PCR reactions, concordant with the multiplex QF-PCR/regression analysis results. (PDF) [file pone.0221227.s010.pdf]
